# Body position and motor imagery strategy effects on imagining gait in healthy adults: Results from a cross-sectional study

**DOI:** 10.1371/journal.pone.0191513

**Published:** 2018-03-15

**Authors:** Olivier Beauchet, Cyrille P. Launay, Harmehr Sekhon, Jennifer Gautier, Julia Chabot, Elise J. Levinoff, Gilles Allali

**Affiliations:** 1 Department of Medicine, Division of Geriatric Medicine, Sir Mortimer B. Davis—Jewish General Hospital and Lady Davis Institute for Medical Research, McGill University, Montreal, Quebec, Canada; 2 Dr. Joseph Kaufmann Chair in Geriatric Medicine, Faculty of Medicine, McGill University, Montreal, Quebec, Canada; 3 Centre of Excellence on Longevity of McGill integrated University Health Network, Quebec, Canada; 4 Service of Geriatric Medicine and Geriatric Rehabilitation, Department of Medicine, Lausanne University Hospital, Lausanne, Switzerland; 5 Department of Neuroscience, Division of Geriatric Medicine, Angers University Hospital, Angers, France; 6 Department of Neurology, Geneva University Hospital and University of Geneva, Geneva, Switzerland; 7 Department of Neurology, Albert Einstein College of Medicine, Yeshiva University, Bronx, New York, United States of America; University of Illinois at Urbana-Champaign, UNITED STATES

## Abstract

**Background:**

Assessment of changes in higher levels of gait control with aging is important to better understand age-related gait instability, with the perspective to improve the screening of individuals at risk for falls. The comparison between actual Timed Up and Go test (aTUG) and its imagined version (iTUG) is a simple clinical way to assess age-related changes in gait control. The modulations of iTUG performances by body positions and motor imagery (MI) strategies with normal aging have not been evaluated yet. This study aims 1) to compare the aTUG time with the iTUG time under different body positions (i.e., sitting, standing or supine) in healthy young and middle age, and older adults, and 2) to examine the associations of body positions and MI strategies (i.e., egocentric versus allocentric) with the time needed to complete the iTUG and the delta TUG time (i.e., relative difference between aTUG and iTUG) while taking into consideration clinical characteristics of participants.

**Methods:**

A total of 60 healthy individuals (30 young and middle age participants 26.6±7.4 years, and 30 old participants 75.0±4.4 years) were recruited in this cross-sectional study. The iTUG was performed while sitting, standing and in supine position. Times of the aTUG, the iTUG under the three body positions, the TUG delta time and the strategies of MI (i.e., ego representation, defined as representation of the location of objects in space relative to the body axes of the self, versus allocentric representation defined as encoding information about body movement with respect to other object, the location of body being defined relative to the location of other objects) were used as outcomes. Age, sex, height, weight, number of drugs taken daily, level of physical activity and prevalence of closed eyes while performing iTUG were recorded.

**Results:**

The aTUG time is significantly greater than iTUG while sitting and standing (P<0.001), except when older participants are standing. A significant difference is reported between iTUG while sitting or standing and iTUG while supine (P≤0.002), higher time being reported in supine position. The multiple linear regressions confirm that the supine position is associated with significant increased iTUG (P≤0.04) and decreased TUG delta time (P≤0.010), regardless of the adjustment. Older participants use the allocentric MI while imagining TUG more frequently than young and middle age participants, regardless of body positions (P≤0.001). Allocentric MI strategy is associated with a significant decrease in iTUG (P = 0.037) only while adjusting for age. A significant increase of iTUG time is associated with age (P≤0.026).

**Conclusions:**

Supine position while imagining TUG represents a more accurate position of actual performance of TUG. Age has a limited effect on iTUG performance but is associated with a change in MI from ego to allocentric representation that decreases the iTUG performances, and thus increases the discrepancy with aTUG.

## Introduction

Motor imagery (MI) is defined as mentally simulating a given action without its execution [1]. MI is used to examine motor control in clinical studies and to identify the functional brain networks involved while performing a motor task in functional brain imaging studies [2–6]. For clinical use, MI performance is assessed using the mental chronometry approach in which the time course of the mental operation is compared to the time course of the performed action [1]. This approach has shown that MI retains many of the properties, in terms of temporal regularities, programming rules and biomechanical constraints, which are observed in the corresponding real action when it comes to execution [1–6]. MI is used as a technique to enhance motor learning and to improve rehabilitation in patients with neurological disorders like stroke [7,8]. More recently, the mental chronometry approach of MI has been used to assess gait impairment in older adults [7]. A better understanding of factors, which may influence MI performance, is required to develop an appropriate clinical test assessing gait impairment in an older population.

The assessment of gait characteristics in older adults has enhanced our understanding of the pathophysiological mechanisms of gait disorders, which is helpful in developing preventive and curative interventions [7–12]. The actual Timed Up and Go (aTUG) test is a broadly used test in geriatric and neurological settings to examine gait impairment [9]. This test provides an objective and standardized gait assessment by measuring the time required for standing up, walking 3 meters, turning, walking back and sitting down [9]. An imagined version of the aTUG, called imagined TUG (iTUG), has been developed to clinically evaluate the highest levels (i.e., cortical) of gait control [7,8,10–12]. An increase in iTUG and an increase in TUG delta time, which is the relative difference between aTUG and iTUG, have been shown with aging, thereby demonstrating that older adults execute the iTUG faster than the aTUG compared to younger adults [8]. This age-related change in performance has been related with an impaired lower-limb proprioception; this impairment being significantly associated with decreased iTUG and increased delta TUG [13]. It has also been suggested that this lower temporal correspondence between executing and imaging gait with aging is related to change in the functional brain networks involved in the gait control [9–12]. All these results underline that the ability to appropriately ambulate in the environment and thus to safely navigate, may be affected with aging as navigation mainly depends on the highest levels of gait control [14,15].

Human navigation depends on both egocentric (i.e., representation of the location of objects in space relative to the body axes of the self) and allocentric (i.e., encoding information about body movement with respect to other object, the location of body being defined relative to the location of other objects) representations of the environment [14,15]. Thus, two main MI related strategies compete to imagine the body’s displacement in the space, which are based on allocentric and egocentric spatial encoding [14–21]. Clinically, it is possible to determine the MI strategies used by asking questions about the engagement of an individual in their gait. For example, do patients observe their environment scrolling while walking (egocentric MI strategy) or do they observe themselves as the person walking (allocentric MI strategy) [14–21]. The influences of both MI strategies on the performances of the iTUG in healthy individuals have not yet been studied.

Previous publications have shown that mental rotation of body parts (i.e., cognitive task in which individuals imagine moving a given body part from its actual posture to that of the same observed or imagined body part may be carried out through a sort of inner simulation [22–25]. These studies underscore that the representation of the body in the brain is continuously updated with regard to peripheral factors such as position or movement of the body. To date, there is no study which has examined the effect of body position on performances of MI of gait.

Normal aging is associated with a decline in gait performance due to changes in the peripheral and central nervous systems [2]. In particular, functional brain imaging studies using MI have reported increased multisensory cortical activation with aging [3–6]. Those results underscore that older adults utilize a gait control, which involves more cognitive resources while imagining compared to young adults. These age-related changes in brain activation have been interpreted as a compensatory mechanism [3,4]. It refers to an additional activation that counteracts age-related decline of brain function and supports successful performance, or as a dedifferentiation mechanism, which reflects age-related difficulties in recruiting specialized neural mechanisms not relevant to task performance [5]. Thus, due to this increased multisensory cortical control of gait that increases with aging and the related central reorganization, it could be suggested that human navigation strategies (i.e., egocentric and allocentric) as well as body positions (i.e., sitting, standing or supine) while imaging TUG may influence the iTUG performance.

Previous results underscored that proprioception influences iTUG performance, suggesting that a condition of realization close to the condition of the actual test could provide similar proprioceptive information and, thus, improve performance. Therefore, we formulated the overall hypothesis that an iTUG condition similar to aTUG (i.e., allocentric MI strategy combined with standing position) could decrease the discrepancy between iTUG and aTUG performances, particularly in older adults compared to younger adults. Thus, we hypothesized, first, that the iTUG time in sitting and supine position but not iTUG time in standing position would be different than the aTUG time. Second, we hypothesized that the lowest TUG delta time could be observed while comparing aTUG and iTUG in standing position. Third, we hypothesized that allocentric strategy and age could be associated with increased iTUG time and decreased TUG delta time (i.e., better concordance between iTUG and aTUG), whereas sitting and supine position would be associated with decreased iTUG time and increased TUG delta time while adjusting on all clinical characteristics of participants. We had the opportunity to test these hypotheses using the baseline assessment of a randomized clinical trial designed to examine the effect of imagined gait on performed gait (trial registration number NCT02120144). This study aims 1) to compare the aTUG time with the iTUG time under different body positions (i.e., sitting, standing or supine) in healthy young and middle age, and older adults, and 2) to examine of the associations of body positions and MI strategies (i.e., egocentric versus allocentric) with the time needed to complete the iTUG and the delta TUG time, while taking into consideration all clinical characteristics of the participants.

## Material and methods

### Participants

Between June to November 2014, 60 individuals (mean age ± standard deviation 50.8±25.1 years with 48.3% women) were recruited in the “Effect of observation plus imagination of gait on stride variability in young and older adults” (OBI) study. Participants were recruited in the community-dwelling population of Angers (France) via poster campaign at Angers University and Angers Health Examination Centers (HEC). This center is a structure of the French health insurance that offers a free and full medical examination. The volunteers returned a phone call to manifest their intention to participate in the study and to evaluate exclusion criteria. The eligibility criteria were: age 20 years and over, adequate understanding of French, absence of cognitive decline (i.e., Mini Mental Status Examination score 30/30) [26] or any neurological and psychiatric diseases, absence of extrapyramidal rigidity of the upper limbs, the presence of severe medical conditions affecting walking, inability to walk 15 minutes unassisted and no acute medical illness in the past month. A total of 30 young and middle age (i.e., aged from 20 to 58 years old) individuals, who were students at Angers University or nurses at Angers university hospital (France), and 30 old (i.e., aged from 70 to 87 years old) healthy individuals without any moderate or severe morbidity were included after having provided their written informed consent.

### Clinical assessment

All included participants underwent a complete clinical examination in the Division of Geriatrics at Angers University Hospital. Information was gathered about age, sex, height (cm), weight (in kg), number of drugs taken daily and physical activity (defined as at least one hour recreational physical activity, such as walking, gymnastics, cycling, swimming or gardening, per week for the past month or more). Body mass index (BMI, in kg/m^2^) was calculated based on anthropometry measurements (i.e., weight in kilograms and height in meters).

### Gait assessment

This study used the TUG test described by Podsiadlo and Richardson [9]. Participants were asked to perform the TUG at their self-selected normal speed in a well-lit environment, using their walking aid if needed. They all completed one trial for both the aTUG and the iTUG in that order: performing the TUG, then, imaging the TUG while sitting in a chair, while standing and while in supine position. The three imagined conditions were performed in a randomized order after the completion of aTUG. Times for each condition were recorded with a stopwatch to the nearest 0.01 second. Before testing, a trained evaluator gave standardized verbal instructions regarding the test procedure. Participants were seated, allowed to use the armrests to stand up and instructed to walk three meters, turn around, walk back to the chair and sit down. The stopwatch was started on the command “ready-set-go” and stopped as the participant sat down. Participants were instructed to imagine TUG and to say “stop” out loud when they were finished. They were asked to describe the strategy used: meaning doing the TUG (egocentric condition) or watching them while performing TUG (allocentric condition). To determine the MI strategy used by the participant two standardized questions with a binary answer (i.e., yes versus no) were asked: 1) Did you engage yourself in the action like seeing the environment scrolling while walking? 2) Have you seen yourself performing the test like observing yourself as a person doing the test? Two concordant answers (i.e., one yes and one no) defined the strategy used. All participants used the same MI strategy, regardless of the body position while imagining TUG. They could choose to do the iTUG with their eyes opened or closed and this condition was recorded. The stopwatch was started on the command “ready-set-go” and stopped when the subject pronounced the word “stop”.

### Outcomes

The main outcome variables were: 1) the mean ± SD of the time to completion for both the aTUG and iTUG while sitting, standing and supine; 2) the mean ± SD of the TUG delta time, which was calculated according to the following the formula: [(aTUG—iTUG) / [(aTUG + iTUG / 2)]] x 100; 3) the type of MI strategy (i.e., egocentric versus allocentric), and 4) the mean ± SD of the participants’ age and the age group category (young and middle age participants versus old participants. In addition, covariates were: sex; the mean ± SD of weight, height, BMI number of drugs taken daily; the prevalence of physically active participants and the prevalence of participants closing their eyes during imagining TUG.

### Ethics

The study was conducted in accordance with the ethical standards set forth in the Helsinki Declaration (1983). Participants in the study were included after obtaining their written informed consent for the study. The Angers local Ethical Committee approved the study protocol.

### Statistics

The participants’ characteristics are summarized using means and standard deviations or frequencies and percentages, as appropriate. For the current analysis, participants are separated into 2 groups based on age, as follows: young and middle age, and old participants. There was no outlier when considering the outcomes. First, in order to examine whether there were any difference between groups for all characteristics, comparisons were performed using unpaired *t*-test, Mann-Whitney or Chi-square test, as appropriate. P-values less than 0.0027 for between-group comparisons are considered as statistically significant because of the multiple comparisons (n = 18). Second, to test the hypothesis that the iTUG time in sitting and supine positions but not in standing position could be different to the aTUG time within each group of participants, intra-group comparisons were performed using paired *t*-test, Wilcoxon test and Friedman test, as appropriate. P-values less than 0.0023 while comparing the aTUG and iTUG times were considered as statistically significant because of the multiple comparisons (n = 21). Third, to test the hypothesis that the lowest TUG delta time could be observed while comparing aTUG and iTUG in standing position, a similar approach was used with P-values less than 0.0041 considered as statistically significant because of the multiple comparisons (n = 12). Fourth, to complete the two previous analysis, a two-way ANOVA with a repeated measure design (iTUG and TUG delta time used as dependent variables in two separated models) and the 3 body positions (standing, sitting, lying) and their interaction term was performed. Fifth, to test the hypothesis that allocentric strategy and age could be associated with increased iTUG time and decreased TUG delta time, whereas sitting and supine positions would be associated with decreased iTUG time and increased TUG delta time, multiple linear regressions were performed. These regression models examined the association between the iTUG (dependent variable) and body positions as well as MI strategies (independent variables) adjusted for participant’s characteristics (i.e., sex, BMI, number of medication taken daily, physical activity and eyes closed). Four models were examined: model 1 examines body positions, MI strategies and age adjusted for the participants’ characteristics (i.e., sex, BMI, number of medication taken daily, physical activity and prevalence of eyes closed) separately, model 2 is adjusted for age (young and middle age participants used as reference) and MI strategies (egocentric representation used as reference), model 3 is adjusted for age (young participants used as reference) and body positions (standing position used as reference), and model 4 is adjusted for age, MI strategies and body positions. All models are adjusted for the participants’ characteristics. Similar analyses have been performed with delta TUG as dependent variable. All statistics were performed using SPSS (version 23.0; SPSS, Inc., Chicago, IL).

## Results

The mean age and the number of drugs taken daily are significantly different between groups; older adults took more drugs compared to younger adults (P**<**0.001, S1 Table). There is a trend for an increased BMI (P = 0.010), aTUG (P = 0.045) and iTUG (P = 0.075) times and a decreased height (P = 0.044) in older compared to young participants. Older participants use the egocentric strategy less frequently compared to their young and middle age counterparts, regardless of body positions (P**≤**0.001). Most of participants keep their eyes open while imagining TUG. They use the same MI strategy for all conditions of iTUG and there is no difference between groups (P = 0.739). As shown in S1 Fig, regardless of the category of participants (i.e., total population, young participants and old participants), aTUG time is significantly longer than iTUG while sitting and standing (P**<**0.001), except when older participants are standing. In this last case, there was only a trend (P = 0.011). There is no significant difference between aTUG and iTUG while supine, as well as, between iTUG while sitting and iTUG while standing, regardless of the group of participants (i.e., total population, young and middle age participants, older participants). A significant difference is reported between iTUG while sitting or standing and iTUG while supine (P≤0.002), higher time being reported in supine position. Comparisons between sitting and standing for delta TUG are non-significant, regardless of the group considered, whereas all other comparisons is significant (P≤0.001; S2 Fig). In addition, the two-way ANOVA with a repeated measure design showed that the body positions have a significant effect on iTUG (F = 21.1 with P<0.001) and TUG delta time (F = 25.3 with P<0.001). There was no interaction between the body positions and age (F = 0.2 with P = 0.830 for iTUG; F = 0.6 with P = 0.529 for delta TUG). As shown in S2 Table, multiple linear regressions show a significant increase of iTUG time with age (P≤0.026). Allocentric MI strategy is associated with significant decrease in iTUG (P = 0.037) while taking into consideration age, whereas there is a trend (P = 0.058) while taking into consideration age and body position. Supine position is associated with an increase in iTUG (P≤0.031), regardless the adjustment. Only supine position is associated with a significant decrease of delta TUG (≤0.010) (S3 Table).

## Discussion

The findings show that iTUG performance is mainly influenced by body position. Supine position is associated with an increased iTUG time and a decreased delta TUG time. In addition, older participants more frequently use the allocentric MI strategy, which is a MI strategy associated with a decreased iTUG time. Furthermore, age is associated with an increased iTUG time. Thus, the best strategy and position, to minimize the discrepancy between aTUG and iTUG, which suggests appropriate gait control, are the egocentric strategy and the supine position, regardless of age.

The study shows a body position-related change in iTUG performance. The supine body position demonstrates to have the closest performance between iTUG and aTUG. This result is unexpected. We hypothesized that the standing position would be the best body position to imaging TUG, as it is the closest position than that used for the condition of aTUG. Our results show the opposite as the supine position is the most distant position of aTUG. Our *a priori* hypothesis is partly supported by our findings: in the comparison between sitting and standing position, the performance during standing position is closer to aTUG than a sitting position. However, the fact that supine position represents the most accurate body position compared to sitting and standing remains difficult to interpret: Additional sensory information due to sitting and standing position may lead to a saturation of the brain’s ability to correctly imagine gait [3,4]. The supine position may be considered as a relative deprivation condition compared to sitting and standing positions when imagined movement is gait, thereby allowing older adults to better focus on gait imagination without the “interference” of afferent sensory signals [4]. This result underlines that the supine position allows a more accurate mental chronometry performance than the other positions, and thus indirectly reinforces the validity of fMRI studies focusing on mental imagery, as participants during fMRI protocols have to remain supine in the scanner. In addition, this result opens the perspective to use the supine position as a reference body position for the assessment of intervention in individuals with mobility impairment in older adults. However, to the best of our knowledge, it is the first time that this result is reported and there were a few number of participants in the study. Thus, there is a need for further studies to confirm this possibility.

Young and old participants differ in their strategy of representation of displacement in the space during iTUG in our study. While the young participants use an egocentric representation, the older prefer the allocentric representation [27]. Safe gait navigation depends on egocentric information about a person’s position relative to the environment as well as allocentric information–which is related to the position of other objects relative to each other in the environment [14–16]. Tolman was the first to link a specific cognitive map to a certain spatial environment (analogous to a cartographic map) such as the position of an object within that environment could be derived from reference to at least two other landmarks [16]. More recently, a broad consensus regarding the involvement of specific brain structures in this cognitive map has been defined [14–29]. Neuropsychological studies of vision and action have demonstrated that egocentric coding is associated with the parietal cortex and that allocentric coding is associated with the temporal cortex [18–21]. It is well established that healthy aging is associated with changes in behavior, neuroanatomical, and functional brain metrics [30,31]. An association between poor gait performance and smaller volume of the parietal lobe was previously found in healthy older adults when using the mean values of gait parameters as the outcomes measures [31–33]. Specifically, both shorter steps and longer double support time were associated with smaller grey matter of the right superior and bilateral inferior parietal lobules. More recently, we demonstrated an association between greater gait stride time variability–a biomarker of cortical gait control—and lower parietal grey matter volume [34]. We can suggest that age-related changes in the parietal cortex alters the regulation of our coordinates in relationship to the surrounding environment with subsequent change in the strategy of displacement representation while iTUG. This “shift” to allocentric representation with aging has important implications in the field of mental imagery: fMRI aging studies focusing on mental imagery of locomotion should include the effect of this “shift” in the interpretation of their findings.

The last result of this study is that aging is only associated with increased iTUG time. This result can be best explained by the fact that the iTUG time depends on the aTUG time. In our study, the aTUG time was greater in older participants compared to younger, therefore the iTUG time was also greater in older participants compared to younger. This closed relationship between aTUG and iTUG times explains the interest of examining delta TUG time, which is relatively different, thus providing an adjustment on mean performance of both TUG conditions [7]. However, as underscored in our results, one limitation in the use of delta TUG time is its higher variability compared to the iTUG time. This point has been previously reported and is related to the way to calculate this outcome [7,8].

This study is not without limitations. First, the small number of participants may limit the generalization of the results. Furthermore, this limitation leads to an inability to compare the MI strategies between the body positions. Second, the participants were not tested for their ability to imagine by a standardized questionnaire like the Vividness of Visual Imagery Questionnaire (VVIQ) [35]. Usually, only individuals with a good ability to imagine (i.e., at least mean rating 3 on the VVIQ rating scale) are included in the study using MI of gait. However, the possible variation of the MI ability in the studied sample is probably low as all participants are healthy. The variation of this ability is caused by diseases [36]. In addition, Berger & Gaunitz [37] underscored that assessing MI ability before a test had no effect on the test performance. Indeed, in their study they showed that participants rated as good imagers did not perform differently from those rated as poor imagers. Third, the age range of young and middle age participants is high: the oldest individual is 58 years old, which is at the border of the World Health Organization’s definition of older adult [38]. However, all the participants are in good health. Fourth, the cross-sectional design of our study does not afford causal inferences. Fifth, we do not control the choice of participants to close their eyes. It has been suggested that imagination improves with eyes closed [6], because it has been reported that eye closure animates the sensory systems [39]. Sixth, participants perform trials as per iTUG condition, which may decrease the statistical power of our study. We made this choice because iTUG is a clinical tool targeting the geriatric population. Thus, worse health conditions of this population are a limitation when repeating the iTUG conditions due to possible exhaustion and attention deficits.

## Conclusion

The results showed significant effects of body positions, MI strategies and age on iTUG performances, body position having the greatest impact. Supine position while imagining TUG represents a more accurate position of actual performance of TUG. Age has a limited effect on iTUG performance but is associated with a change in MI from ego to allocentric representation, which decreases the iTUG performances, and thus increases the discrepancy with aTUG.

## Supporting information

S1 Table Characteristics of participants separated into 2 groups according to their age (n = 60). (DOCX)

S2 Table Multiple linear regressions showing the association between the iTUG (dependent variable) and body positions as well as motor imagery strategies (independent variables) adjusted for participant’s characteristics (n = 60). (DOCX)

S3 Table Multiple linear regressions showing the association between the delta TUG. * (dependent variable) and body positions as well as motor imagery strategies (independent variables) adjusted for participant’s characteristics (n = 60).
(DOCX)

S1 Fig Comparison of the Timed Up and Go performances under different body positions and categories of populations (n = 60). *: Intra-group comparisons based on Friedman test; †: Intra-group comparisons based on paired-*t*-test; P significant <0.0023 due to the multiple comparisons (n = 21).
(TIF)

S2 Fig Comparison of the delta time Timed Up and Go under different body positions and categories of populations (n = 60). *: Intra-group comparisons based on Friedman test; †: Intra-group comparisons based on paired-*t*-test; P significant <0.0041 due to the multiple comparisons (n = 12).
(TIF)

S1 File S1_OBI_DataBase_Width_Excel. (XLSX)
