# Supplementary material for: Body position and motor imagery strategy effects on imagining gait in healthy adults: Results from a cross-sectional study
Source: PLoS One. 2018 Mar 15;13(3):e0191513. doi: 10.1371/journal.pone.0191513 (PMC5854233; doi:10.1371/journal.pone.0191513)
Supplement: S1 Table — (DOCX) [file pone.0191513.s001.docx]

**S1 Table. Characteristics of participants separated into 2 groups according to their age (n=60)**

|  | Total population  (n=60) | Participants | | P-Value‡ |
| --- | --- | --- | --- | --- |
|  |  | Young and middle age*  (n=30) | Old†  (n=30) |  |
| Age (years), mean ± SD | 50.8±25.1 | 26.6±7.4 | 75.0±4.4 | **<0.001** |
| Female, n (%) | 29 (48.3) | 17 (56.7) | 12 (40.0) | 0.196 |
| Weight (kg), mean ± SD | 67.3±11.4 | 66.4±12.0 | 68.3±10.9 | 0.522 |
| Height (cm), mean ± SD | 168.2±9.9 | 170.7±9.4 | 165.6±9.7 | 0.044 |
| Body mass index (kg/m2), mean ± SD | 23.8±3.3 | 22.7±3.3 | 24.8±3.0 | 0.010 |
| Number of drugs daily taken, mean ± SD | 1.4±1.6 | 0.6±0.9 | 2.2±1.8 | **<0.001** |
| Physically active, n (%) | 21 (35.0) | 18 (60.0) | 21 (70.0) | 0.417 |
| Timed Up & Go test (second), mean ± SD |  |  |  |  |
| Performed | 9.2±1.7 | 8.7±1.4 | 9.6±1.8 | 0.045 |
| Imagined while sitting | 7.7±2.4 | 7.1±2.2 | 8.2±2.6 | 0.075 |
| Imagined while standing | 7.9±2.7 | 7.3±2.5 | 8.6±2.7 | 0.165 |
| Imagined while supine | 9.1±3.3 | 8.5±2.9 | 9.7±3.6 | 0.289 |
| Delta¶ Timed Up & Go test (%), mean ± SD |  |  |  |  |
| Sitting | 20.5±21.0 | 23.4±22.3 | 17.6±19.6 | 0.289 |
| Standing | 17.8±23.8 | 22.3±26.7 | 13.2±19.8 | 0.140 |
| Supine | 5.8±28.9 | 7.8±29.4 | 3.8±28.8 | 0.600 |
| Egocentric strategy§, n (%) |  |  |  |  |
| Sitting | 20 (33.3) | 16 (53.3) | 4 (13.3) | **0.001** |
| Standing | 20 (33.3) | 16 (53.3) | 4 (13.3) | **0.001** |
| Supine | 24 (40.0) | 21 (70.0) | 3 (10.0) | **<0.001** |
| Eyes closed#, n (%) | 11 (18.3) | 5 (16.7) | 6 (20.0) | 0.739 |

n: number of participants; *: participants aged from 20 to 58 years; †: participants aged from 70 to 87 years; ‡: Comparison based on unpaired t-test or Chi-square test, as appropriate; ¶: Calculated from the formula: delta time = [(aTUG–iTUG)/(aTUG+iTUG)/2] x100; §: encoding information about body movement with respect to other object, the location of body being defined relative to the location of other objects); #: participants used the same eyes strategy for all iTUG conditions; P significant (i.e., <0.0027 due to multiple comparisons n=18) indicated in bold.
