# Supplementary material for: Body position and motor imagery strategy effects on imagining gait in healthy adults: Results from a cross-sectional study
Source: PLoS One. 2018 Mar 15;13(3):e0191513. doi: 10.1371/journal.pone.0191513 (PMC5854233; doi:10.1371/journal.pone.0191513)
Supplement: S2 Table — (DOCX) [file pone.0191513.s002.docx]

**S2 Table. Multiple linear regressions showing the association between the iTUG (dependent variable) and body positions as well as motor imagery strategies (independent variables) adjusted for participant’s characteristics (n=60).**

|  | | Model 1 |  | Model 2 |  | Model 3 |  | Model 4 |
| --- | --- | --- | --- | --- | --- | --- | --- | --- |
|  |  | β [95%CI] P-value |  | β [95%CI] P-value |  | β [95%CI] P-value |  | β [95%CI] P-value |
| Age | |  |  |  |  |  |  |  |
| Young and middle age | | Ref. |  | Ref. |  | Ref. |  | Ref. |
| Old | | 1.12  [0.14;2.10]  **0.026** |  | 1.52  [0.48;2.56]  **0.005** |  | 1.12  [0.16;2.08]  **0.023** |  | 1.48  [0.45;2.50]  **0.005** |
| Motor imagery strategies | |  |  |  |  |  |  |  |
| Egocentric* | | Ref. |  | Ref. |  | - |  | Ref. |
| Allocentric† | | -0.54  [-1.49;0.41]  0.266 |  | -1.07  [-2.06;-0.07]  **0.037** |  | - |  | -0.95  [-1.94;0.03]  0.058 |
| Body position | |  |  |  |  |  |  |  |
| Standing | | Ref. |  | - |  | Ref. |  | Ref. |
| Sitting | | -0.26  [-1.24;0.72]  0.604 |  | - |  | -0.26  [-1.23;0.71]  0.600 |  | -0.26  [-1.22;0.70]  0.597 |
| Supine | 1.13  [0.15;2.11]  **0.024** | |  | - |  | 1.13  [0.16;2.10]  **0.023** |  | 1.06  [0.10;2.03]  **0.031** |

β: Coefficient of regression beta corresponding to an increase or a decrease in imagined Timed up and go time; CI: confidence interval; ref: reference level; Model 1: Separated model for age, motor imagery strategy and body position; Model 2 adjusted for age and MI strategies (allo versus ergocentric representation); Model 3 adjusted for age and body positions (i.e; standing, sitting and supine); Model 4 adjusted for age, MI strategies and body positions; all models are adjusted for sex, body mass index, number of medication taken daily, physical activity and prevalence of eyes closed; * representation of the location of objects in space relative to the body axes of the self; †: encoding information about body movement with respect to other object, the location of body being defined relative to the location of other objects)
