# Supplementary material for: Body position and motor imagery strategy effects on imagining gait in healthy adults: Results from a cross-sectional study
Source: PLoS One. 2018 Mar 15;13(3):e0191513. doi: 10.1371/journal.pone.0191513 (PMC5854233; doi:10.1371/journal.pone.0191513)
Supplement: S3 Table — * (dependent variable) and body positions as well as motor imagery strategies (independent variables) adjusted for participant’s characteristics (n = 60). (DOCX) [file pone.0191513.s003.docx]

**S3 Table. Multiple linear regressions showing the association between the delta TUG* (dependent variable) and body positions as well as motor imagery strategies (independent variables) adjusted for participant’s characteristics (n=60).**

|  | | Model 1 |  | | Model 2 | |  | | Model 3 | |  | | Model 4 | |  |
| --- | --- | --- | --- | --- | --- | --- | --- | --- | --- | --- | --- | --- | --- | --- | --- |
|  |  | β [95%CI] P-value |  | | β [95%CI] P-value | |  | | β [95%CI] P-value | |  | | β [95%CI] P-value | |  |
| Age | |  |  | |  | |  | |  | |  | |  | |  |
| Young and middle age | | Ref. |  | | Ref. | |  | | Ref. | |  | | Ref. | |  |
| Old | | -6.64  [-15.60;2.31]  0.145 |  | | -9.09  [-18.67;0.49]  0.063 | |  | | -6.64  [-15.34;2.05]  0.133 | |  | | -8.64  [-17.96;0.69]  0.069 | |  |
| Motor imagery strategies | |  |  | |  | |  | |  | |  | |  | |  |
| Egocentric† | | Ref. |  | | Ref. | |  | | - | |  | | Ref. | |  |
| Allocentric‡ | | 3.34  [-5.29;11.97]  0.446 |  | | 6.49  [-2.70;15.68]  0.165 | |  | | - | |  | | 5.29  [-3.68;14.26]  0.246 | |  |
| Body position | |  |  | |  | |  | |  | |  | |  | |  |
| Standing | | Ref. |  | | - | |  | | Ref. | |  | | Ref. | |  |
| Sitting | | 2.76  [-6.03;11.56]  0.536 |  | | - | |  | | 2.76  [-6.00;11.52]  0.534 | |  | | 2.76  [-5.99;11.51]  0.534 | |  |
| Supine | -11.95  [-20.74;-3.16]  **0.008** | | |  | | - | |  | | -11.95  [-20.71;-3.19]  **0.008** | |  | | -11.60  [-20.37;-2.83]  **0.010** | |

β: Coefficient of regression beta corresponding to an increase or a decrease in delta Timed up and go time; CI: confidence interval; ref: reference level; Model 1: Separated model for age, motor imagery strategy and body position; Model 2 adjusted for age and MI strategies (allo versus egocentric representations); Model 3 adjusted for age and body positions (i.e; standing, sitting and supine); Model 4 adjusted for age, MI strategies and body positions; all models are adjusted for sex, body mass index, number of medication taken daily, physical activity and prevalence of eyes closed; *: Calculated from the formula: delta time = [(aTUG–iTUG)/(aTUG+iTUG)/2] x100; †: representation of the location of objects in space relative to the body axes of the self; ‡: encoding information about body movement with respect to other object, the location of body being defined relative to the location of other objects)
