# Supplementary figures and images for: Body position and motor imagery strategy effects on imagining gait in healthy adults: Results from a cross-sectional study
Source: PLoS One. 2018 Mar 15;13(3):e0191513. doi: 10.1371/journal.pone.0191513 (PMC5854233; doi:10.1371/journal.pone.0191513)

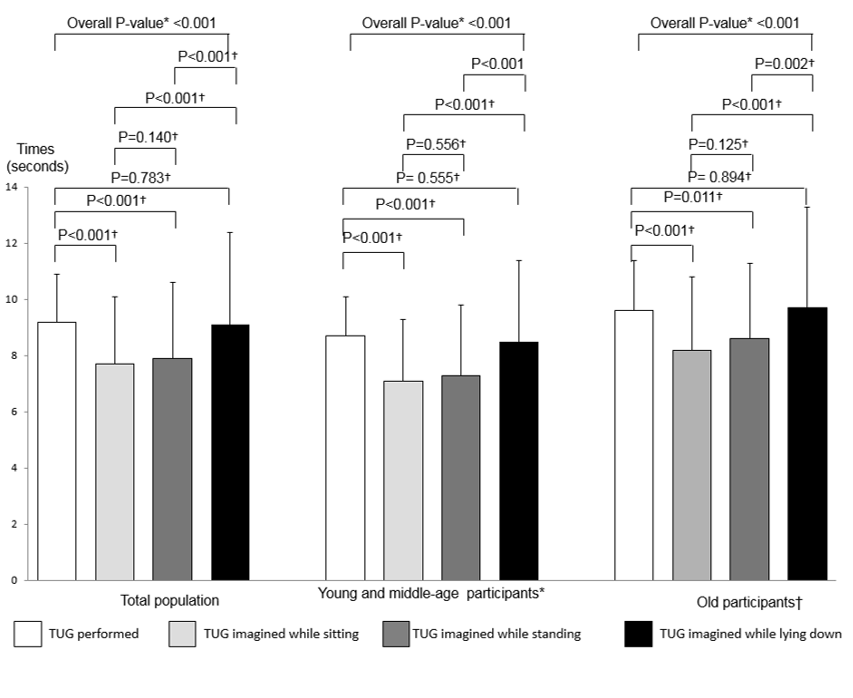

Supplement: S1 Fig — *: Intra-group comparisons based on Friedman test; †: Intra-group comparisons based on paired-t-test; P significant <0.0023 due to the multiple comparisons (n = 21). (TIF) [file pone.0191513.s004.tif]

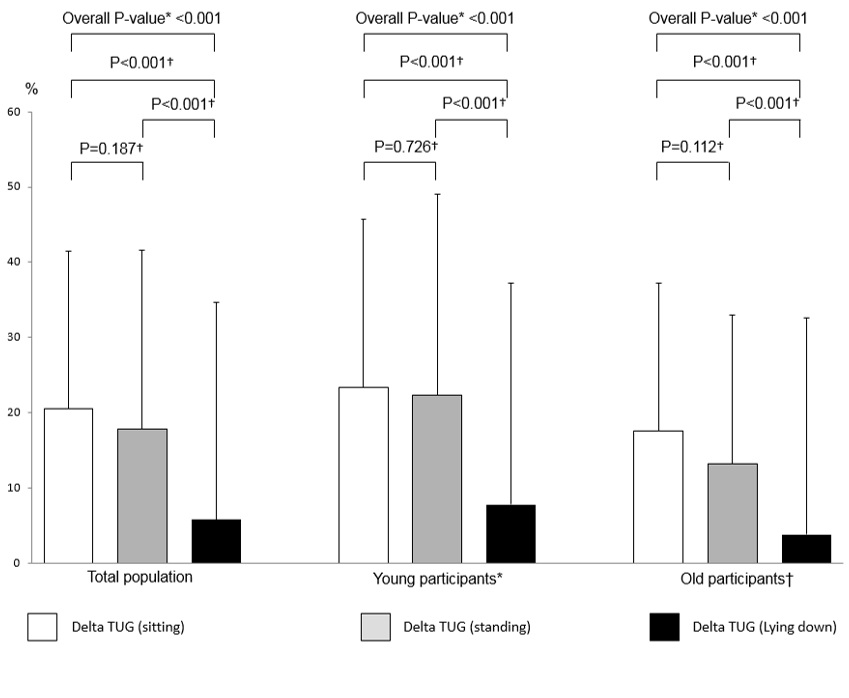

Supplement: S2 Fig — *: Intra-group comparisons based on Friedman test; †: Intra-group comparisons based on paired-t-test; P significant <0.0041 due to the multiple comparisons (n = 12). (TIF) [file pone.0191513.s005.tif]
